# Alterations in circulating levels of vitamin D binding protein, total and bioavailability of vitamin D in diabetic retinopathy patients

**DOI:** 10.1186/s12902-022-01084-6

**Published:** 2022-07-01

**Authors:** Zhila Maghbooli, Sepideh Ebrahimi Meimand, Ali-Asghar Malek Hosseini, Arash Shirvani

**Affiliations:** 1 Multiple Sclerosis Research Center, Neuroscience Institute, Tehran University of Medical Sciences, Tehran, Iran; 2 Tehran University of Medical Sciences, Tehran, Iran; 3 Endocrinology and Metabolism Research Institute, Tehran University of Medical Sciences, Tehran, Iran

**Keywords:** Type 2 Diabetes mellitus, Retinopathy, Vitamin D binding protein, Vitamin D, Bioavailability of vitamin D

## Abstract

**Aims:**

This study aimed to investigate the association between circulating levels of vitamin D binding protein (VDBP) and its genotypes and diabetic retinopathy risk.

**Methods:**

This case–control study recruited 154 patients with type 2 diabetes mellitus; 62 with diabetic retinopathy (DR) and 92 without DR and diabetic nephropathy (DN). Circulating levels of 25-hydroxyvitamin D3 and VDBP levels were measured in the patients. The genotype and phenotype of VDBP were evaluated based on two common VDBP variations; rs7041 and rs4588.

**Results:**

Serum levels of VDBP were significantly lower in patients with DR than in patients without DR and/or DN (Ln-VDBP (μg/ml): 6.14 ± 0.92 vs. 6.73 ± 1.45, *p* = 0.001) even after adjustment for age, sex, body mass index, disease duration, estimated glomerular filtration rate (eGFR), HbA1C, insulin therapy profile, and serum levels of 25(OH)D. The distribution of VDBP phenotypes and genotypes in the two studied groups were nearly the same, and the distribution was similar to that of the general population.

**Conclusions:**

In this study, we found the association between lower circulating levels of VDBP and risk of DR. However, the precise mechanism linking these two remains unknown. Further and more in-depth research is needed to find out the underlying causes of the relationship.

**Supplementary Information:**

The online version contains supplementary material available at 10.1186/s12902-022-01084-6.

## Introduction

Diabetic retinopathy (DR) is one of the most common etiologies for visual loss in the population aged 20–64 [1]. It is the first etiology of preventable blindness in the working-age (20–45 years) population in developed countries, and the estimated prevalence of DR is 18.45% [2–5]. Poor glycemic control and disease duration are the major risk factors for DR. However, a disturbance of metabolic factors like vitamin D deficiency increases the risk of developing DR [6–8]. Vitamin D may play a part in the pathogenic mechanisms predisposing to complications of diabetes via the dysregulation of glucose homeostasis, modulating insulin resistance, and pancreatic β-cell function [9, 10].

A meta-analysis has reported a lack of enough evidence supporting the efficacy of vitamin D supplementation in complications of type 2 diabetes and stated that vitamin D supplementation has no proven effect on fasting glucose levels, impaired glucose tolerance, improving insulin resistance, and glycemic control in diabetic patients [11]. On the other hand, it has been reported that moderate to high doses of vitamin D may reduce the risk of cardiovascular disease [12]. Indeed, vitamin D supplementation may reduce the risk of progression from prediabetes to diabetes [13]. These discrepancies may be caused by vitamin D binding protein (VDBP) dysregulation in diabetes patients.

VDBP, the key determinant of vitamin D, can efficiently affect the sustainability, bioavailability, and biological performance of vitamin D. Vitamin D and its metabolites should pass the cell membrane by forming a complex with VDBP to have their function and attach to the nuclear vitamin D receptor [14]. VDBP (P02774), a member of the albumin family, is a polymorphic protein known as the main carrier for active metabolites of vitamin D [15, 16]. It is synthesized in the liver and its concentration remains stable during life comparatively. However, liver failure, proteinuria, and malnutrition lead to lower circulating levels of VDBP [17]. VDBP has a high affinity to the principal vitamin D metabolites; around 85% of circulating 25(OH) D and 1, 25(OH) 2D carries via binding to VDBP with high affinity, and approximately 15% of them are attached to albumin and less than 0.4% of them are free [18, 19]. The bioavailability of 25(OH)D can be regulated through VDBP variations resulting in different phenotypes and consequently different circulating levels of total VDBP [20]. It can be inferred that metabolic differences due to racial variations of VDBP may be the underlying cause of the association between 25(OH)D and diabetes and its microvascular complications. The total 25(OH)D level, as a single marker, is not sufficient to evaluate vitamin D status accurately [21]. In cases with an altered circulation of VDBP, measuring VDBP and calculating bioavailable 25(OH)D may help to determine vitamin D status more accurately [21].

In this study, we evaluated the hypothesis that VDBP has an important role in the abruption of vitamin D homeostasis in diabetic patients with microvascular complications. We investigated whether there is an association between serum VDBP and bioavailable 25(OH)D with diabetic retinopathy and assessed the risk association of VDBP polymorphisms and diabetic retinopathy.

## Subjects, materials, and methods

### Study population

Between July 2012 and September 2013, 154 patients with the diagnosis of T2DM from a referral diabetes clinic affiliated with Tehran University of medical sciences (Tehran, Iran) were recruited for this case–control study; 62 of them had retinopathy and 92 of them did not have retinopathy or nephropathy (control group).

As mentioned previously, the inclusion was based on American diabetes association (ADA) criteria for type 2 diabetes, which consists of “a fasting blood glucose ≥ 126 mg/dL on two separate occasions, random (non-fasting) blood glucose ≥ 200 mg/dL on two separate occasions or a blood glucose > 200 mg/dL at 2 h during a standard oral glucose tolerance” [22]. History of acute or chronic inflammatory or autoimmune disorders, being a current smoker, and pregnancy at the time of study in both groups were the primary exclusion criteria.

### Retinopathy and nephropathy definitions

The retinopathy and nephropathy diagnosis criteria were described previously [4, 23]. Briefly, “an eye examination was conducted for every participant by an ophthalmologist, after dilating the pupils with eye drops. Furthermore, a slit lamp and indirect ophthalmoscopy were used for retina examination. For at-risk patients, fluorescein angiography was also performed”.

A urine albumin-to-creatinine ratio was obtained from a random urine collection of all patients and classified as normal (urine microalbumin: creatinine ratio ≤ 30 μg/mg), microalbuminuria (urine microalbumin: creatinine ratio > 30 μg/mg and ≤ 299 μg/mg) and macroalbuminuria (urine microalbumin: creatinine ratio ≥ 300 μg/mg) at least in two distinct samples.

The control participants were diabetic patients with normal urine albumin values and no established retinopathy.

### Biochemical measurements

Patients had overnight fasting for 10–14 h before taking blood samples. The separated sera of the participants were kept at -80 °C before the analysis. The serum levels of following items were measured through an enzymatic colorimetric assay [Pars-Asmun kits, Iran] and using an auto-analyzer (Hitachi 902, Japan) with intra-assay precision of 0.63% to 1.23% and inter-assay precision of 1.09% to 1.8%): fasting glucose (normal range: 72–126 mg/dl), total cholesterol (TC) (normal cutoff < 200 mg/dl), high-density lipoprotein (HDL) cholesterol (normal cutoff: ≥ 40 mg/dl for women, ≥ 35 mg/dl for men), and low-density lipoprotein (LDL) cholesterol (normal cutoff: < 100 mg/dl), triglycerides (TG) (normal cutoff < 150 mg/dl), blood urea nitrogen (BUN) (normal range: 1.9–3 mg/dl), creatinine (Cr) (normal range: 0.6–1.3 mg/dl), uric-acid (normal range: 3.5–7.2 mg/dl), and albumin (normal range: 3.4–5.5 g/dl). The same auto analyzer was administered to measure Urine microalbumin and creatinine levels with normal urine microalbumin: creatinine ratio ≤ 30 μg/mg.

Estimated glomerular filtration rate (eGFR) was calculated based on the Chronic Kidney Disease Epidemiology Collaboration (CKD-EPI) equation; for Cr > 61.9 μmol/l [if female]: GFR = 144 × (Scr/61.9)-1.209 × (0.993)Age, for Cr > 79.6 μmol/l [if male] GFR = 141 × (Scr/79.6)-1.209 × (0.993)Age [24]. In our population study, serum Cr levels were over 61.9 μmol/l in women and over 79.6 μmol/l in men.

Ion exchange chromatography with a DS5 set (DREW, UK) was used for measuring glycated hemoglobin (HbA1c) levels. To assess the glycemic control status in the patients, the mean HbA1c > 7% was considered poorly controlled diabetes [25].

The serum insulin level was measured by an immunoenzymometric assay [Monobind Inc., USA]. The intra-assay and inter-assay coefficients of variation (CVs) for insulin were 5.9% and 9.2%, respectively. For radioimmunoassay measurement of Serum 25(OH)D, the Biosource kit (Biosource Europe SA, Belgium) was used with intra-assay and inter-assay coefficients of variation (CV) were 5.2% and 7.5%, respectively. Serum levels of vitamin D less than 30 ng/ml were considered vitamin D deficiency or insufficiency [26].

Parathyroid hormone (PTH) was measured by quantitative two-step sandwich immunoassay using the Chemiluminescent Microparticle Immuno technique (Architect intact PTH, 8K25, Germany) with a sensitivity of ≤ 5 pg/ml at a total CV of 20% and cross-reactivity of ≤ 0.01. The cut-off level of 65 pg/ml was considered a maximum normal level of PTH [27].

To measure VDBP serum levels, Polyclonal ELIZA kit (Mybiosource, MBS564062) was administered based on the manufacturer’s instructions; inter-assay precision: CV < 10% and intra-assay precision: CV < 8%. The vitamin D bioavailability was estimated using serum levels of vitamin D, VDBP and albumin according to the equation “[Bio D] = [DFree] + [DAlb] = (Kalb· [Alb] + 1) · [DFree]” [28, 29]. The term "bioavailable 25(OH)D" refers to “both genotype-independent and genotype-specific bioavailable 25(OH)D” in this study.

### Genotyping and phenotyping

The extraction of DNA, genotyping, and phenotyping of VDBP have been described in our previous study [30].

Polymerase chain reaction (PCR), and restriction fragment length polymorphism analysis (RFLP) were administered to determine the polymorphisms of VDBP in exon 11; rs7041 at codon 416 (Asp → Glu), and rs4588 at codon 420 (Thr → Lys). According to RFLP, rs4588 genotypes named CC (Thr/ Thr) (809 bp), AA (Lys/Lys) (584 and 225 bp), AC (Lys/Thr) (584, 225, and 809 bp); and rs7041 genotypes marked as GG (Glu/Glu) (577 and 232 bp), TT (Asp/Asp) (nondigested bond at 809), and TG (Asp/Glu) (577, 232, and 809 bp). Ten percent of the PCR samples were sequenced directly to confirm the result of PCR–RFLP.

According to VDBP genotypes, three common variants of VDBP were determined, considering no human being possesses the combination of rs4588 (T) and rs7041(C): Gc1F (rs7041 (A) and rs4588 (G)), Gc1S (GC1S = rs7041(C) and rs4588 (G)), and Gc2 (GC2 = rs7041 (A) and rs4588 (T)). These genotypes result in six different recognized phenotypes: 1 s/1 s, 1 s/1f, 1 s/2, 1f/1f, 1f/2, and 2/2.

### Statistical analysis

The analysis was conducted by SPSS statistical software (version 20) and the expression of data was in form of the number and percentage for categorical values and the mean ± standard deviation for continuous variables. Analysis of data normality was accomplished through the Shapiro–Wilk test. The distribution of circulating levels of VDBP, PTH, insulin, and BUN were not normal. To correct their normality distribution a natural Log transformation was applied. The Student’s t-test and Chi-square test for continuous variables and categorical variables (control as a reference group), respectively, were used to compare comparisons between the study groups. In the case of vitamin D bioavailability, the data did not follow a normal distribution. It was presented in median and interquartile ranges and the Mann-Whiney U test was used to compare the differences between the case and control groups.

Assessment of the Hardy–Weinberg equilibrium (HWE) and linkage disequilibrium (LD) tests of SNP was also conducted. Notably, the participants’ demographics and clinical features were reported using descriptive statistics. The genotype and allele frequencies as well as phenotype analysis were performed by Chi-square and Fisher exact tests, respectively. Pearson correlation was used to consider the relationship between circulating levels of VDBP, vitamin D, and PTH.

To examine the association between VDBP and DR risk, the multivariable logistic regression model was used. Confounding factors were selected based on the parameters associated with DR, including age, sex, body mass index (BMI), insulin therapy profile, physical activity, sampling seasons (winter), vitamin D supplementation, vitamin D levels, poor control of glycemia, kidney dysfunction, and the disease duration. If a *P*-value was less than 0.2 (*P*-value < 0.2), it was adjusted in multivariable logistic regression analyses. All tests were two-sided, and *P*-values less than 0.05 were considered significant.

## Results

### Baseline characteristics

A total number of 154 diabetic patients participated in this case–control study; 62 patients were diagnosed with DR and 92 of them had no distinguished DR and/or DN (control group). The age and sex ratio were nearly the same in the two groups, but DR group patients were older and disease duration was longer in them (Table 1). In addition, the percentage of patients being treated with insulin in the DR group was greater than the control group (37.1% vs. 20.7%, respectively, *p* = 0.02). More details on the characteristics of the participants are available in Table S1. There were no significant differences in circulating levels of FBS, TG, LDL, and TC. Patients with DR had significantly lower levels of HDL even after adjustment for age, sex, and disease duration (*p* = 0.001). In the course of poor control diabetes (HbA1c > 7%), both groups were nearly the same; %60.9 in the DR group vs %69.4 in the control group (*p*-value = 0.3).

**Table 1:** Demographic characteristic and biochemical analysis in diabetic patients with and without retinopathy

Demographic characteristic	Diabetic Mellitus with retinopathy (*N* = 62)	Diabetic Mellitus without retinopathy and/or nephropathy (*N* = 92)	*p*-value
Age (years)^a^	59.6 ± 6.0	55.0 ± 6.0	0.001
Sex ( Women)^c^	27 (43.5%)	53 (57.6%)	0.1
BMI (kg/m^2^)^a^	28.9 ± 4.6	29.0 ± 5.6	0.8
Disease duration (year)^a^	16.5 ± 6.7	10.1 ± 6.2	0.001
Vitamin D deficiency or insufficiency (< 30 ng/ml)	51 (83.6%)	70 (77.8%)	0.4
**Laboratory outcomes**			
FBS (mg/dL)^a^	150.1 ± 64.4	143.6 ± 46.2	0.45
Ln BUN (mg/dL)^a^	3.1 ± 0.4	2.7 ± 0.3	0.001
Uric acid (mg/dL)^a^	5.62 ± 1.57	5.10 ± 1.33	0.02
TG (mg/dL)^a^	133.42 ± 68.05	157.82 ± 94.17	0.05
TC (mg/dL)^a^	155.67 ± 40.71	152.82 ± 36.76	0.6
HDL (mg/dL)^a^	42.19 ± 10.13	49.00 ± 10.33	0.001
LDL (mg/dL)^a^	86.25 ± 25.91	81.56 ± 23.82	0.23
eGFR (mL/min)^a^	69.80 ± 30.10	92.69 ± 27.11	0.001
Cr (mg/dL)^a^	1.5 ± 0.9	1.1 ± 0.5	0.001
Albumin (g/dL)^a^	5.07 ± 0.55	5.26 ± 0.96	0.1
Ln Insulin ( (μU/L)^a^	2.45 ± 0.93	2.27 ± 0.65	0.2
Urine microalbumin (mg/L)^a^	184.11 ± 45.87	8.36 ± 0.97	0.001
25(OH)D (ng/mL)^a^	18.5 ± 11.6	21.4 ± 10.2	0.08
Ln PTH (pg/mL)^a^	3.8 ± 0.6	3.5 ± 0.4	0.01
Vitamin D bioavailability (ng/ml)^b^	1.7 (3.04)	0.86 (2.2)	0.03

*BMI* Body mass index, *BUN* Blood urea nitrogen, *Cr* Creatinine, *eGFR* Estimating glomerular filtration rate, *FBS* Fasting blood serum, *HDL* High-density lipoprotein, *LDL* Low-density lipoprotein, *Ln* Natural logarithm, *PTH* Parathyroid hormone

^a^mean ± SD

^b^median (IQR)

^c^Number (percentage)

For patients who were not under insulin therapy in each study group, there was no significant difference in the serum levels of insulin between the DR and control groups (Ln-insulin (μUI/l): 2.21 ± 0.6 vs. 2.40 ± 0.9; respectively, *p* = 0.3).

Regarding kidney function, serum levels of BUN, uric acid, and Cr and urine levels of microalbumin were significantly higher in the DR patients. Consequently, the eGFR levels were significantly lower in DR patients compared to diabetic patients without microvascular complications.

### Serum levels of vitamin D, vitamin D binding protein, and bioavailability of vitamin D

In the total study population, 9.8% of patients took vitamin D supplementation. The mean serum levels of vitamin D were 20.9 ± 9.6 ng/ml and only 19% of patients had sufficient circulating levels of vitamin D (≥ 30 ng/ml) (Table 1).

In the course of diabetic retinopathy, the prevalence of vitamin D deficiency/or insufficiency was nearly the same in both groups, DR and control groups; 83.6% vs. 77.8%, respectively (*p* = 0.4).

The serum levels of VDBP were significantly lower in the DR group compared control group (Ln- VDBP (μg/ml): 6.14 ± 0.92 in the DR group vs. 6.76 ± 1.51 in the control group, *p* = 0.002).

In the DR group, there was a significant negative correlation between circulating levels of VDBP and vitamin D (*r* = -0.3, *p* = 0.02) but not with PTH levels (*p* = 0.2). In the control group, there were no significant correlations between circulating levels of VDBP and vitamin D (*p* = 0.4) and PTH (*p* = 0.3).

As DR patients were older and had a longer duration of diabetes, and lower eGFR and serum levels of vitamin D, the logistic regression model was used to minimize their effects. After adjustment for age, sex, insulin therapy profile, eGFR, vitamin D levels, and the disease duration, there was an independent association between lower levels of VDBP and DR risk (Beta = 0.7, 95% CI lower–upper: 0.5–0.9, *p* = 0.04).

**Fig. 1:**
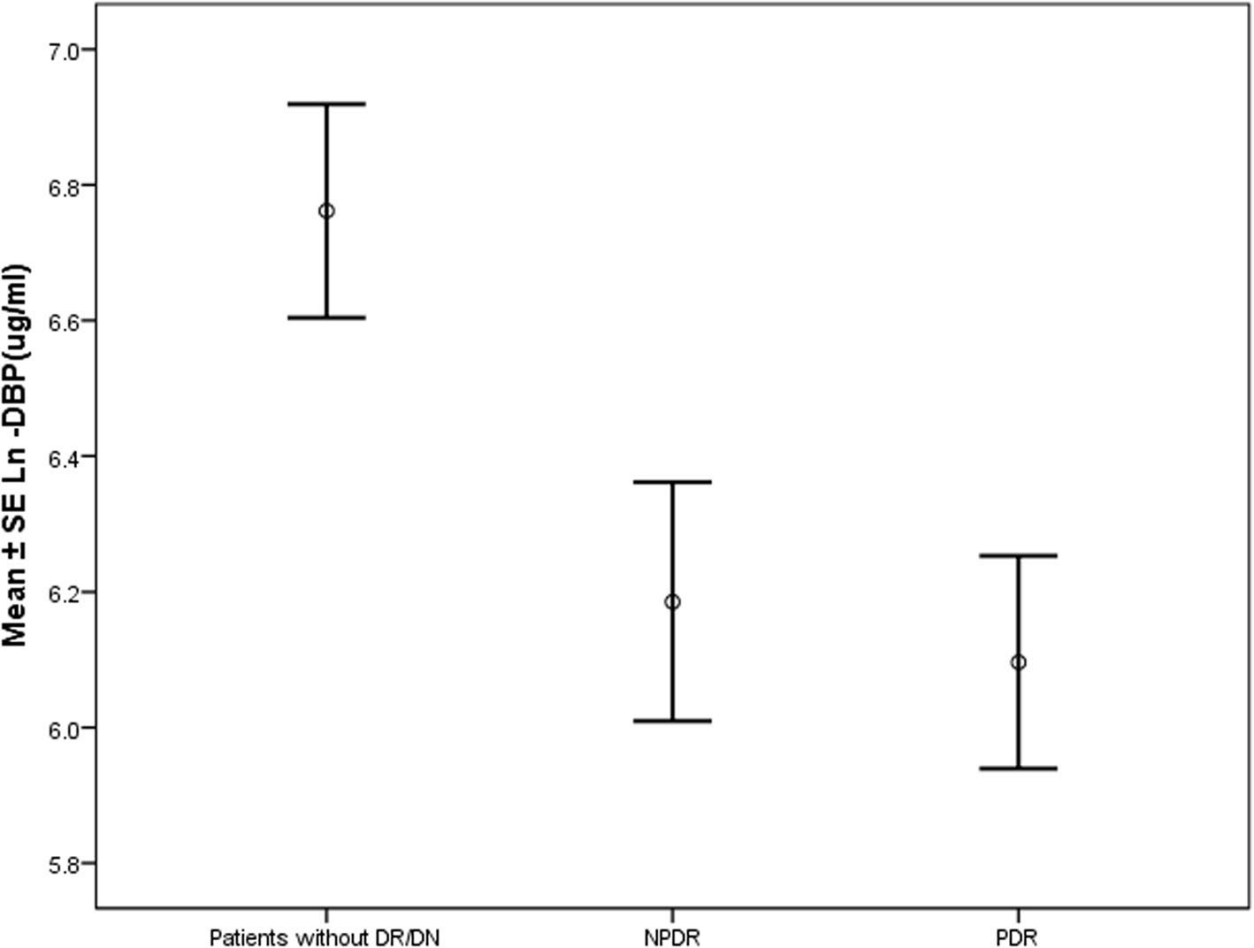
Comparison of VDBP levels between proliferative retinopathy, non-proliferative diabetic retinopathy, and no retinopathy in diabetic patients

In the subgroup analysis of the DR group, the VDBP levels were approximately similar in patients with proliferative (PDR) and non-proliferative diabetic retinopathy (NPDR) (*p* = 0.9) (Fig. 1).

There was a decreasing trend in circulating levels of VDBP in relation to retinopathy progression; (LnVDBP (ug/ml): with PDR, 6.1 ± 0.8; NPDR, 6.2 ± 0.8; and without PDR or NPDR, 6.7 ± 1.5 (*p* = 0.01)). There was no significant difference in the circulating levels of VDBP between the PDR and NPDR subgroups (*p* = 0.7) (Fig. 1).

The estimations of vitamin D bioavailability showed higher rates in the DR group than in the control group (median (IQR): 1.7 (3.04) ng/ml vs. 0.86 (2.2), *p* = 0.03). There was no significant constant association between vitamin D bioavailability and DR after adjusting the confounder factors including age, sex, eGFR, and disease duration (*p* = 0.14).

### Polymorphisms in the vitamin D-binding protein gene

Genotyping was performed in 59 diabetic patients with retinopathy and 81 diabetic patients without retinopathy (control group) for rs4588 and rs7041 of the VDBP gene. Table 2 presents the distribution of genotype, allele, and phenotype frequency in the DR and control groups. The genotypes based on both SNPs were consistent with Hardy–Weinberg equilibrium in the DR group (*p* = 0.96 for rs4588, *p* = 0.16 for rs7041) and the control group (*p* = 0.33 for rs4588, *p* = 0.14 for rs7041).

**Table 2:** Genotype, allele frequency, and phenotype of Vitamin D Binding Protein gene in diabetic patients with and without retinopathy

	Diabetic mellitus with retinopathy (*N* = 59)	Diabetic mellitus without retinopathy and/or nephropathy (*N* = 81)	*P*-value
Genotype- rs4588			
**CC** (Thr/Thr)	54.2%(32)	64.2%(52)	0.4
AA (Lys/Lys)	6.8%(4)	6.2%(5)	
AC (Lys/Thr)	39.0%(23)	29.6%(24)	
Genotype- rs7041			
**TT** (Asp/Asp)	8.5%(5)	8.6%(7)	0.9
GG (Glu/Glu)	37.3%(22)	38.3%(31)	
TG (Asp/Glu)	54.2%(32)	53.1%(43)	
allele Freq. rs4588			
**C**	0.74	0.75	0.3
A	0.26	0.25	
allele Freq.rs7041			
**T**	0.29	0.29	0.3
G	0.71	0.71	
GC phenotype			
Gc1f, Gc1f	1.7%(1)	1.2% (1)	0.7^a^
Gc1s, Gc1s	37.3%(22)	38.3%(31)	
Gc2, Gc2	1.7%(1)	1.2%(1)	
GC1f,GC1s	15.3%(9)	24.7%(20)	
GC1f,GC2	6.8%(4)	7.4%(6)	
GC1s,GC2	37.3%(22)	27.2%(22)	

^a^fisher’s exact test

In both groups, the minor-allele frequency for SNP rs4588 was “A” and the dominant genotype was recognized as “CC”. In both groups, the minor-allele frequency for SNP rs7041 was T and the dominant genotype was recognized as the heterozygous TG. The frequencies of the genotypes according to both SNPs, rs4588, and rs7041, were nearly the same between the two groups; DR and control (*P* > 0.05). There was no significant difference in the frequency of minor-alleles of SNPs, rs4588 and rs7041, between the two studied groups (*P* > 0.05). Table 2 shows the distribution of the six possible haplotypes according to the two variants in both groups. The GC2-GC2 phenotype was similar in two groups; 1.7% in the DR group and 1.2% in the control group.

## Discussion

In this study, we considered serum levels of vitamin D, VDBP and vitamin D bioavailability in patients with DR. Our data showed that serum VDBP levels were lower in DR patients than those without DR and/or DN.

A few studies considered circulating levels of VDBP in diabetes and its complications [31, 32]. Fawzy and AlSel evaluated the VDBP levels in the serum and urine of diabetes patients and reported significantly elevated urine VDBP levels in correlation with the degree of albuminuria. Moreover, the authors showed that serum VDBP was also higher in patients with microalbuminuria or macroalbuminuria in comparison with patients who had normal levels of urine albumin. Another study conducted by Tian et al. presented elevated urine VDBP levels as a biomarker for DN [32]. Elevated levels of urine VDBP were demonstrated in diabetic patients previously, which is likely to be associated with tubule-interstitial dysfunction in patients with nephropathy and nephrotic syndrome [33–35]. In the normal kidney, to prevent the loss of vitamin D in the urine, VDBP bounds to 25OHD and other vitamin D metabolites to reabsorb them.

In the current study, we measured serum levels of VDBP that showed a negative correlation with microalbuminuria in both groups. In our study population, DR patients were older and had a longer duration of diabetes and lower eGFR compared with diabetic patients without DR. Consequently, DR patients with tubular dysfunction lose more VDBP through urine excretion and have lower circulating levels of VDBP. Grymonprez et al. showed that there is a negative correlation between serum VDBP levels and urine VDBP levels [36]. Consequently, tubular dysfunction can be considered the main reason for lower serum VDBP levels in diabetes patients.

One of the molecular mechanisms that explain the lower serum VDBP levels in DR patients with tubular dysfunction is megalin/Cubilin receptors. Megalin is a big protein receptor in the membrane that belongs to low-density lipoprotein receptors [37] and is expressed in the epithelial cells of the proximal tubule in the kidney [38]. Cubilin is known as a co-receptor of megalin that binds and internalizes many small proteins, including VDBP, retinol-binding protein, and transcobalamin [37]. The VDBP bounded to 25-OH vitamin D transports freely through the glomerulus to the proximal tubules and then the complex is reabsorbed by megalin and its co-receptor [39]. The reabsorption by megalin facilitates the activity of 1α-hydroxylase in the epithelial cells of the proximal tubule to produce 1, 25-dihydroxy vitamin D [40, 41]. A previous study by Thrailkill et al. showed that urinary excretion of megalin is enhanced in patients with microalbuminuria due to diabetes type 1 [42]. In vivo studies reported vitamin D deficiency, and enhanced urinary excretion of VDBP in mouse models [41, 43]. Enhanced urinary excretion of VDBP and 25(OH)D and also decreased expression of megalin in the kidneys have been reported in animal models with diabetes [44, 45].

There is a hypothesis suggesting that lower levels of serum VDBP can be secondary to megalin deficiency in diabetic patients with tubular dysfunction [46]. Megalin is also expressed in other tissues including the lung, uterus, and eye [47]. It is detected in the outermost layer of the retina and ciliary body of the eye [48]. The role of megalin in the development of the normal eye seems to be essential, but the details of the process have remained unknown to date. Furthermore, our analysis showed that there was an association between serum VDBP and DR, independent of vitamin D levels and eGFR. It is likely; that the lower levels of serum VDBP in DR patients may have a pathological role in developing DR through megalin function in retinal cells.

Another mechanism that could explain the possible role of serum VDBP in developing DR is PTH imbalance due to vitamin D deficiency. Growing evidence shows that low concentrations of 1, 25-dihydroxy vitamin D are correlated with the risk of DR and other complications of diabetes [49, 50]. However, the marked deficiency in our population may have confounded the study. In our study, the prevalence of vitamin D deficiency or insufficiency (25(OH)D < 30 ng/mL) was over 70% in both groups. Although a decreased circulating level of vitamin D is a trigger of compensatory increased PTH, it seems that the setpoint is different in DR patients. Our data showed that higher PTH levels are more common in DR patients compared with diabetic patients without microvascular complications (DR and/or DN), which is in agreement with the results of other studies [51, 52]. The higher levels of PTH as a compensatory mechanism of vitamin D deficiency in diabetic patients can stimulate inflammatory cytokines like tumor necrosis factor (TNF)-α and interleukin (IL)-6 that have important roles in the pathogenesis of DR [53, 54]. In line with our study, some studies have mentioned that circulating levels of VDBP are lower in some inflammatory diseases [30, 55]. As DR has inflammatory pathogenesis [56], it suggests that chronic PTH excess in diabetic patients may have indirect effects on circulating levels of VDBP by modulating inflammatory markers.

Regarding vitamin D bioavailability, our data showed higher rates in the DR group than in the control group, but the association disappeared after adjustment for confounder factors including, age, sex, insulin therapy profile, eGFR, and disease duration. As expected, lower VDBP concentrations led to lower VDBP-bound 25(OH) D levels and higher vitamin D bioavailability (non-bound vitamin D) among the DR patients. The longer disease duration and tubular dysfunction of DR patients make them more vulnerable to impairment of 1,25-dihydroxy vitamin D reabsorption in the kidney.

Although VDBP is known as a vitamin D carrier, only 5% of its function is allocated to transporting vitamin D metabolites to its target cells [57]. VDBP binds to actin and it can depolymerize polymeric actin [58]. Monomeric actin easily turns into polymeric form in the plasma and results in clogging of the microvascular system, such as retinal microvessels. VDBP prevents polymerization and subsequent clogging [59]. It is worth mentioning that in our data, the DR patients were older and had higher disease duration. By modeling, we tried to minimize the effect of the main confounding factors. Nevertheless, even after adjusting for eGFR, age, sex, insulin therapy profile, and the disease duration, there was an independent association between lower circulating VDBP levels and DR. Indeed, there was a decreasing trend in circulating levels of VDBP in terms of progressing retinopathy; PDR and NPDR, but not statistically significant.

It points out a new concern that circulating levels of VDBP may have a causality role in the development of retinopathy. One of the important functional roles of VDBP is activating macrophages [60]. Macrophage activation may be associated with DR throughout several mechanisms. Macrophage adhesion to the capillary endothelium can result in capillary occlusion and finally retinal ischemia [61, 62] by stimulation of cytokines [63–68]. TNF-α is a cytokine produced by macrophages that has an important role in DR pathogenesis [63]. It increases the permeability of retinal endothelium through downregulation of tight junction proteins' expression. Increased endothelial permeability may result in rupture of the blood-retinal barrier [64]. TNF-α is also a chemoattractant of leukocytes, which can also stimulate leukocyte adhesion and cause oxidation and production of reactive oxygen species as a result of optic nerve degeneration and retinal ganglion cells' death [65, 66]. TNF-α levels have been increased in animal models of proliferative retinopathy due to retinal neovascularization [66]. IL-1β is another cytokine mainly synthesized by macrophages. It can induce angiogenesis and ocular neovascularization [67]. The role of IL-1β in DR has been demonstrated in diabetic mouse models [68]. Taken together, macrophage activation may mediate the association of VDBP with DR.

VDBP genetic variations were also considered in metabolic disorders like diabetes [69–72]. The VDBP gene is located on chromosome 4q12; which is a region that seems to be associated with plasma glucose and insulin concentrations in Pima Indians [9]. In this study, we have also investigated the two common missense VDBP polymorphisms, named rs7041 and rs8455, Based on the two SNPs, three major alleles have been recognized for the VDBP gene (GC1F, GC1S, GC2) [73]. The difference between GC1 and GC2 is due to alterations of four amino acids; 152 Gly / Glu, 311 Glu / Arg, 416 Asp / Glu, and 420 Arg / Thr [74]. The difference between GC1S and GC1F is subsequent to the substitution of aspartic acid by glutamic acid at position 416 [75]. There is controversy about the correlation between VDBP genotype polymorphisms and susceptibility to type 2 diabetes mellitus. Some studies have suggested a moderate correlation in Asians but no correlation in Caucasians [69, 70]. Szathmary et al. reported that homozygotes for Gc 1F-1F had lower fasting insulin among other phenotypes [71]. However, Wei-Zhen et al. have reported that the distribution of genotypes according to both codons and phenotypes was approximately similar in healthy controls and diabetic patients [72]. The results of genotyping and phenotyping of VDBP showed no difference between the two studied groups. The distribution of phenotypes and genotypes according to the variants of the two determined SNPs (rs4588, rs7041) in both groups was similar to the general population. Our findings are in line with the results of previous studies that have shown no correlation between the variants of these two codons with the risk of DR.

However, this study has some limitations. Firstly, a case–control study could not show that variation in the circulating levels of VDBP is a consequence or its underlying cause of retinal or kidney damage in those patients. Therefore, further longitudinal studies will be conducted to investigate the VDBP roles in diabetic patients during the progression of DR. Secondly, we did not investigate the association between VDBP and DR to figure out as a marker of microangiopathy in diabetes patients. Further studies need to consider VDBP accompanied by other inflammatory markers that are involved in diabetic microangiopathy.

Finally, we did not measure urine VDBP levels and we could not conclude the exact association between microalbuminuria and urine VDBP concentration. It can be very useful for determining the role of VDBP and the more definite reason behind these findings.

Taken together, the results of this study could be beneficial in further identifying the role of VDBP in diabetic retinopathy. VDBP is a small protein from the albuminoid gene family, and its size is smaller than albumin and lost more by urinary excretion due to tubular dysfunction. Consequently, in future studies, it is possible to consider VDBP as a biomarker for the progression of microvascular complications.

## Conclusion

We can conclude from the findings of this study that VDBP phenotypes and genotypes based on the rs4588 and rs7041 codons are not associated with diabetic retinopathy. VDBP levels are lower in diabetic patients with retinopathy, which may be a result of urinary excretion of VDBP due to tubular dysfunction in diabetic patients. There is another possibility that lower levels of VDBP result in the progression of diabetic retinopathy by clogging retinal microvessels. Further investigations are needed to understand the mechanism relating VDBP to diabetic retinopathy.

## Supplementary Information

**Additional file 1.** Clinical characteristics, and environmental factors.

## Data Availability

The datasets generated and/or analyzed during the current study are available in the European Variation Archive (https://www.ebi.ac.uk/eva/?Submit-Data) repository, with accession number # 10,743,821.

## References

[1] Chatziralli IP, Sergentanis TN, Keryttopoulos P, Vatkalis N, Agorastos A, Papazisis L (2010). Risk factors associated with diabetic retinopathy in patients with diabetes mellitus type 2. BMC Res Notes.

[2] Song P, Yu J, Chan KY, Theodoratou E, Rudan I (2018). Prevalence, risk factors and burden of diabetic retinopathy in China: a systematic review and meta-analysis. J Glob Health.

[3] Antonetti DA, Klein R, Gardner TW (2012). Diabetic retinopathy. N Engl J Med.

[4] Yau JW, Rogers SL, Kawasaki R, Lamoureux EL, Kowalski JW, Bek T, Chen SJ, Dekker JM, Fletcher A, Grauslund J (2012). Global prevalence and major risk factors of diabetic retinopathy. Diabetes Care.

[5] Wong TY, Cheung CM, Larsen M, Sharma S, Simo R (2016). Diabetic retinopathy. Nat Rev Dis primers.

[6] Lu L, Lu Q, Chen W, Li J, Li C, Zheng Z (2018). Vitamin D(3) Protects against Diabetic Retinopathy by Inhibiting High-Glucose-Induced Activation of the ROS/TXNIP/NLRP3 Inflammasome Pathway. J Diabetes Res.

[7] Luo BA, Gao F, Qin LL (2017). The Association between Vitamin D Deficiency and Diabetic Retinopathy in Type 2 Diabetes: A Meta-Analysis of Observational Studies. Nutrients.

[8] Yuan J, Zhou JB, Zhao W, Zhang RH, Cai YH, Shu LP, Qi L, Yang JK (2019). Could Vitamin D be Associated with Proliferative Diabetic Retinopathy? Evidence from Pooling Studies. Horm Metab Res.

[9] Baier LJ, Dobberfuhl AM, Pratley RE, Hanson RL, Bogardus C (1998). Variations in the vitamin D-binding protein (Gc locus) are associated with oral glucose tolerance in nondiabetic Pima Indians. J Clin Endocrinol Metab.

[10] Parveen R, Kapur P, Venkatesh S, Agarwal NB (2019). Attenuated serum 25-hydroxyvitamin D and vitamin D binding protein associated with cognitive impairment in patients with type 2 diabetes. Diabetes Metab Syndr Obes.

[11] George PS, Pearson ER, Witham MD (2012). Effect of vitamin D supplementation on glycaemic control and insulin resistance: a systematic review and meta-analysis. Diabetic Med.

[12] Wang L, Manson JE, Song Y, Sesso HD (2010). Systematic review: Vitamin D and calcium supplementation in prevention of cardiovascular events. Ann Intern Med.

[13] Pittas AG, Jorde R, Kawahara T, Dawson-Hughes B (2020). Vitamin D Supplementation for Prevention of Type 2 Diabetes Mellitus: To D or Not to D?. J Clin Endocrinol Metab.

[14] Jorde R (2019). The Role of Vitamin D Binding Protein, Total and Free 25-Hydroxyvitamin D in Diabetes. Front Endocrinol (Lausanne).

[15] Blanton D, Han Z, Bierschenk L, Linga-Reddy MV, Wang H, Clare-Salzler M, Haller M, Schatz D, Myhr C, She JX (2011). Reduced serum vitamin D-binding protein levels are associated with type 1 diabetes. Diabetes.

[16] Moon M, Song H, Hong HJ, Nam DW, Cha MY, Oh MS, Yu J, Ryu H, Mook-Jung I (2013). Vitamin D-binding protein interacts with Abeta and suppresses Abeta-mediated pathology. Cell Death Differ.

[17] Bikle DD, Gee E, Halloran B, Haddad JG (1984). Free 1,25-dihydroxyvitamin D levels in serum from normal subjects, pregnant subjects, and subjects with liver disease. J Clin Invest.

[18] Bouillon R, Van Baelen H, De Moor P (1977). 25-Hydroxyvitamin D and its Binding Protein in Maternal and Cord Serum. J Clin Endocrinol Metab.

[19] Bouillon R, Van Assche FA, Van Baelen H, Heyns W, De Moor P (1981). Influence of the Vitamin D-binding Protein on the Serum Concentration of 1,25-Dihydroxyvitamin D3: SIGNIFICANCE OF THE FREE 1,25-DIHYDROXYVITAMIN D3 CONCENTRATION. J Clin Investig.

[20] Brink M, Johansson L, Nygren E, Ärlestig L, Hultdin J, Rantapää-Dahlqvist S (2018). Vitamin D in individuals before onset of rheumatoid arthritis-relation to vitamin D binding protein and its associated genetic variants. BMC rheumatology.

[21] Kim HJ, Ji M, Song J, Moon HW, Hur M, Yun YM (2017). Clinical Utility of Measurement of Vitamin D-Binding Protein and Calculation of Bioavailable Vitamin D in Assessment of Vitamin D Status. Ann Lab Med.

[22] Maghbooli Z, Hossein-nezhad A, Larijani B, Amini M, Keshtkar A (2015). Global DNA methylation as a possible biomarker for diabetic retinopathy. Diabetes Metab Res Rev.

[23] Maghbooli Z, Shabani P, Gorgani-Firuzjaee S, Hossein-Nezhad A (2016). The association between bone turnover markers and microvascular complications of type 2 diabetes. J Diabetes Metab Disord.

[24] Inker LA, Astor BC, Fox CH, Isakova T, Lash JP, Peralta CA, Kurella Tamura M, Feldman HI (2014). KDOQI US commentary on the 2012 KDIGO clinical practice guideline for the evaluation and management of CKD. Am J Kidney Dis.

[25] Vasudevan AR, Ghosh S, Srivastava R, Premawardhana LD (2003). Low HbA1c levels in a poorly controlled diabetic. Postgrad Med J.

[26] Holick MF, Binkley NC, Bischoff-Ferrari HA, Gordon CM, Hanley DA, Heaney RP, Murad MH, Weaver CM (2011). Evaluation, treatment, and prevention of vitamin D deficiency: an Endocrine Society clinical practice guideline. J Clin Endocrinol Metab.

[27] Pagana K, Pagana T, Pagana T (2019). Mosby's Diagnostic and Laboratory Test Reference.

[28] Powe CE, Evans MK, Wenger J, Zonderman AB, Berg AH, Nalls M, Tamez H, Zhang D, Bhan I, Karumanchi SA (2013). Vitamin D-Binding Protein and Vitamin D Status of Black Americans and White Americans. N Engl J Med.

[29] Arnaud J, Constans J (1993). Affinity differences for vitamin D metabolites associated with the genetic isoforms of the human serum carrier protein (DBP). Hum Genet.

[30] Maghbooli Z, Omidifar A, Varzandi T, Salehnezhad T, Sahraian MA (2021). Reduction in circulating vitamin D binding protein in patients with multiple sclerosis. BMC Neurol.

[31] Fawzy MS, Abu AlSel BT (2018). Assessment of Vitamin D-Binding Protein and Early Prediction of Nephropathy in Type 2 Saudi Diabetic Patients. J Diabetes Res.

[32] Tian XQ, Zhao LM, Ge JP, Zhang Y, Xu YC (2014). Elevated urinary level of vitamin D-binding protein as a novel biomarker for diabetic nephropathy. Exp Ther Med.

[33] Rao PV, Lu X, Standley M, Pattee P, Neelima G, Girisesh G, Dakshinamurthy K, Roberts CT, Nagalla SR (2007). Proteomic identification of urinary biomarkers of diabetic nephropathy. Diabetes Care.

[34] Bennett MR, Pordal A, Haffner C, Pleasant L, Ma Q, Devarajan P (2016). Urinary Vitamin D-Binding Protein as a Biomarker of Steroid-Resistant Nephrotic Syndrome. Biomarker Insights.

[35] Sato KA, Gray RW, Lemann J (1982). Urinary excretion of 25-hydroxyvitamin D in health and the nephrotic syndrome. J Lab Clin Med.

[36] Grymonprez A, Proesmans W, Van Dyck M, Jans I, Goos G, Bouillon R (1995). Vitamin D metabolites in childhood nephrotic syndrome. Pediatric nephrology (Berlin, Germany).

[37] Bouillon R, Schuit F, Antonio L, Rastinejad F (2020). Vitamin D Binding Protein: A Historic Overview. Front Endocrinol (Lausanne).

[38] Christensen EI, Birn H (2002). Megalin and cubilin: multifunctional endocytic receptors. Nat Rev Mol Cell Biol.

[39] Mahadevappa R, Nielsen R, Christensen EI, Birn H (2014). Megalin in acute kidney injury: foe and friend. Am J Physiol Renal Physiol.

[40] Negri AL (2006). Proximal tubule endocytic apparatus as the specific renal uptake mechanism for vitamin D-binding protein/25-(OH)D3 complex. Nephrology (Carlton).

[41] Nykjaer A, Dragun D, Walther D, Vorum H, Jacobsen C, Herz J, Melsen F, Christensen EI, Willnow TE (1999). An endocytic pathway essential for renal uptake and activation of the steroid 25-(OH) vitamin D3. Cell.

[42] Thrailkill KM, Nimmo T, Bunn RC, Cockrell GE, Moreau CS, Mackintosh S, Edmondson RD, Fowlkes JL (2009). Microalbuminuria in type 1 diabetes is associated with enhanced excretion of the endocytic multiligand receptors megalin and cubilin. Diabetes Care.

[43] Leheste JR, Melsen F, Wellner M, Jansen P, Schlichting U, Renner-Müller I, Andreassen TT, Wolf E, Bachmann S, Nykjaer A (2003). Hypocalcemia and osteopathy in mice with kidney-specific megalin gene defect. FASEB J.

[44] Anderson RL, Ternes SB, Strand KA, Rowling MJ (2010). Vitamin D homeostasis is compromised due to increased urinary excretion of the 25-hydroxycholecalciferol-vitamin D-binding protein complex in the Zucker diabetic fatty rat. Am J Physiol Endocrinol Metab.

[45] Fowlkes J, Bunn R, Cockrell G, Clark L, Wahl E, Lumpkin C, Thrailkill K (2011). Dysregulation of the Intrarenal Vitamin D Endocytic Pathway in a Nephropathy-Prone Mouse Model of Type 1 Diabetes. Exp Diabetes Res.

[46] Thrailkill KM, Jo CH, Cockrell GE, Moreau CS, Fowlkes JL (2011). Enhanced excretion of vitamin D binding protein in type 1 diabetes: a role in vitamin D deficiency?. J Clin Endocrinol Metab.

[47] Fisher CE, Howie SEM (2006). The role of megalin (LRP-2/Gp330) during development. Dev Biol.

[48] Zheng G, Bachinsky DR, Stamenkovic I, Strickland DK, Brown D, Andres G, McCluskey RT (1994). Organ distribution in rats of two members of the low-density lipoprotein receptor gene family, gp330 and LRP/alpha 2MR, and the receptor-associated protein (RAP). J Histochem Cytochem.

[49] Aksoy H, Akçay F, Kurtul N, Baykal O, Avci B (2000). Serum 1, 25 dihydroxy vitamin D (1, 25 (OH) 2D3), 25 hydroxy vitamin D (25 (OH) D) and parathormone levels in diabetic retinopathy. Clin Biochem.

[50] Reheem RNAMA, Fattah MAHMA (2013). Serum vitamin D and parathormone (PTH) concentrations as predictors of the development and severity of diabetic retinopathy. Alexandria J Med.

[51] Suzuki A, Kotake M, Ono Y, Kato T, Oda N, Hayakawa N, Hashimoto S, Itoh M. Hypovitaminosis D in type 2 diabetes mellitus: association with microvascular complications and type of treatment. Endocrine J. 2006:53(4):503–10. 10.1507/endocrj.k06-001 16829706

[52] Aksoy H, Akçay F, Kurtul N, Baykal O, Avci B (2000). Serum 1,25 dihydroxy vitamin D (1,25(OH)2D3), 25 hydroxy vitamin D (25(OH)D) and parathormone levels in diabetic retinopathy. Clin Biochem.

[53] Taverna MJ, Selam J-L, Slama G (2005). Association between a protein polymorphism in the start codon of the vitamin D receptor gene and severe diabetic retinopathy in C-peptide-negative type 1 diabetes. J Clin Endocrinol Metab.

[54] Mitnick MA, Grey A, Masiukiewicz U, Bartkiewicz M, Rios-Velez L, Friedman S, Xu L, Horowitz MC, Insogna K (2001). Parathyroid hormone induces hepatic production of bioactive interleukin-6 and its soluble receptor. Am J Physiol Endocrinol Metab.

[55] Nyomba B, Bouillon R, Bidingija M, Kandjingu K (1986). Moor Pd: Vitamin D metabolites and their binding protein in adult diabetic patients. Diabetes.

[56] Rübsam A, Parikh S, Fort PE (2018). Role of inflammation in diabetic retinopathy. Int J Mol Sci.

[57] Yamamoto N, Delves PJ (1998). Vitamin D and the Immune System. Encyclopedia of Immunology.

[58] Bouillon R, Pauwels S: Chapter 7 - The Vitamin D-Binding Protein. In: Vitamin D (Fourth Edition). edn. Edited by Feldman D: Academic Press; 2018: 97–115.

[59] Baelen H, Bouillon R, Moor P (1980). Vitamin D-binding protein (Gc-Globulin) binds actin. J Biol Chem.

[60] Nagasawa H, Uto Y, Sasaki H, Okamura N, Murakami A, Kubo S, Kirk KL, Hori H (2005). Gc protein (vitamin D-binding protein): Gc genotyping and GcMAF precursor activity. Anticancer Res.

[61] Murugeswari P, Shukla D, Rajendran A, Kim R, Namperumalsamy P, Muthukkaruppan V (2008). Proinflammatory cytokines and angiogenic and anti-angiogenic factors in vitreous of patients with proliferative diabetic retinopathy and eales' disease. Retina (Philadelphia, Pa).

[62] Ghasemi H, Ghazanfari T, Yaraee R, Faghihzadeh S, Hassan ZM (2011). Roles of IL-8 in ocular inflammations: a review. Ocul Immunol Inflamm.

[63] Parameswaran N, Patial S (2010). Tumor necrosis factor-α signaling in macrophages. Crit Rev Eukaryot Gene Expr.

[64] Aveleira CA, Lin CM, Abcouwer SF, Ambrósio AF, Antonetti DA (2010). TNF-α signals through PKCζ/NF-κB to alter the tight junction complex and increase retinal endothelial cell permeability. Diabetes.

[65] Madigan MC, Sadun AA, Rao NS, Dugel PU, Tenhula WN, Gill PS (1996). Tumor necrosis factor-alpha (TNF-alpha)-induced optic neuropathy in rabbits. Neurol Res.

[66] Majka S, McGuire PG, Das A (2002). Regulation of matrix metalloproteinase expression by tumor necrosis factor in a murine model of retinal neovascularization. Invest Ophthalmol Vis Sci.

[67] Guarda G, So A (2010). Regulation of inflammasome activity. Immunology.

[68] Vincent JA, Mohr S (2007). Inhibition of caspase-1/interleukin-1beta signaling prevents degeneration of retinal capillaries in diabetes and galactosemia. Diabetes.

[69] Iqbal K, Islam N, Azam I, Asghar A, Mehboobali N, Iqbal MP (2017). Association of Vitamin D binding protein polymorphism with risk of type 2 diabetes mellitus in a Pakistani urban population: A case control study. JPMA J Pak Med Assoc.

[70] Wang G, Li Y, Li L, Yu F, Cui L, Ba Y, Li W, Wang C (2014). Association of the vitamin D binding protein polymorphisms with the risk of type 2 diabetes mellitus: a meta-analysis. BMJ Open.

[71] Szathmary EJE (1987). The effect of Gc genotype on fasting insulin level in Dogrib Indians. Hum Genet.

[72] Ye W-Z, Dubois-Laforgue D, Bellanné-Chantelot C, Timsit J, Velho G (2001). Variations in the vitamin D-binding protein (Gc locus) and risk of type 2 diabetes mellitus in French Caucasians. Metabolism - Clinical and Experimental.

[73] Miller JR, Lechler PJ, Mackin G, Germanoski D, Villarroel LF (2007). Evaluation of particle dispersal from mining and milling operations using lead isotopic fingerprinting techniques, Rio Pilcomayo Basin. Bolivia Science of The Total Environment.

[74] Braun A, Bichlmaier R, Cleve H (1992). Molecular analysis of the gene for the human vitamin-D-binding protein (group-specific component): allelic differences of the common genetic GC types. Hum Genet.

[75] Ravnsborg T, Olsen DT, Thysen AH, Christiansen M, Houen G, Højrup P (2010). The glycosylation and characterization of the candidate Gc macrophage activating factor. Biochim Biophys Acta.

