# Supplementary material for: Alterations in circulating levels of vitamin D binding protein, total and bioavailability of vitamin D in diabetic retinopathy patients
Source: BMC Endocr Disord. 2022 Jul 1;22:169. doi: 10.1186/s12902-022-01084-6 (PMC9250226; doi:10.1186/s12902-022-01084-6)
Supplement: Supplementary file 1 — Additional file 1. Clinical characteristics, and environmental factors. [file 12902_2022_1084_MOESM1_ESM.docx]

**Clinical characteristics, and environmental factors:**

The clinical characteristic, diabetes risk factors, and environmental risk factors of two groups; diabetes patients with DR and the control group without DR/DN are presented in Table S1.

Diabetes risk factors were defined based on ADA 2022 [1]. Hypertension was defined in participants with a BP ≥140/90 mm Hg or current use of high blood pressure medications. Dyslipidemia was defined as TG >250 mg/dL and/or HDL < 35 mg/dL or using lipid-lowering medications. Obesity was classified based on BMI≥30 kg/m^2^[2].

In our study population, 83.8% of diabetes patients had dyslipidemia and 69.5% had hypertension. Also,

39% of diabetes patients were obese. However, the prevalence of diabetes risk factors was similar in both groups; diabetes patients with DR and the control group without DR/DN.

Further analysis of the data showed no significant differences in the prevalence of macrovascular complications like the history of coronary artery disease (CAD) and cerebrovascular accident (CVA).

Based on anti-diabetes treatments, drugs were classified into four groups: thiazolidinediones, metformin, sulfonylureas, and insulin injection. Most of the study population took combinations of drugs; in the total population, 63.6% of diabetes patients took at least two types of anti-diabetes drugs.

As the data present in table S1, patients in the DR group were more under insulin therapy than the control group (p=0.02).

Taking vitamin D and calcium supplementations were similar in both groups; case and control. Physical activity was classified as follows: vigorous (30 min, at least 5 days per week), moderate (30 min, 3–4 days per week), low (10–30 min, less than 3 days per week) activates, and no activity (only daily activity). The data analysis shows no significant differences in the two groups; case and control groups.

Sampling seasons were classified based on winter (late November, December, January, and February).

**Table S1:** Diabetes risk factors and macrovascular complications in patients with retinopathy and control group without retinopathy and nephropathy.

|  | **Diabetes patients**  **with DR (N=62)** | **Diabetes patients**  **without DR/DN (N=92)** | **P-value** |
| --- | --- | --- | --- |
| **Taking anti-diabetic drug at the sampling time** | | | |
| No treatment | 11(17.7%) | 1 (1.1%) | 0.000 |
| Insulin injection | 23 (37.1%) | 19 (20.7%) | 0.02 |
| Metformin | 35 (56.5%) | 86 (93.5%) | 0.000 |
| Thiazolidinedione | 7 (11.3%) | 9 (9.8%) | 0.7 |
| Sulfonylureas | 22 (35.5%) | 53 (57.6%) | 0.007 |
| Diabetes Risk factors |  |  |  |
| hypertension | 46 (74.2%) | 61 (66.3%) | 0.29 |
| Dyslipidemia | 81 (77.4%) | 81 (88.0%) | 0.08 |
| Obesity | 25 (40.3%) | 35 (38.0%) | 0.77 |
| **Environmental risk factors** | | | |
| Vitamin D supplementation | 6 (9.7%) | 9 (9.8%) | 0.98 |
| Calcium supplementation | 7 (11.3%) | 16 (17.4%) | 0.29 |
| Physical activity |  |  |  |
| Vigorous activity | 25 (40.3%) | 31 (33.7%) | 0.84 |
| Moderate activity | 10 (16.1%) | 18 (19.6%) |  |
| Low activity | 12 (19.4%) | 18 (19.6%) |  |
| No activity | 15 (24.2%) | 25 (27.2%) |  |
| Sampling season ( Winter) | 21(33.9%) | 25 (27.2%) | 0.4 |
| **Macro-vascular complications** | | | |
| coronary artery disease | 12 (19.4%) | 15 (16.3%) | 0.62 |
| cerebrovascular accident | 4 (6.5%) | 1(1.1%) | 0.15* |

*Fisher exact test

Reference:

1. Association AD: **Standards of Medical Care in Diabetes—2022 Abridged for Primary Care Providers**. *Clinical Diabetes* 2022, **40**(1):10-38.

2. Weir CB, Jan A: **BMI classification percentile and cut off points**. 2019.
